# Multifaceted bioactivity of brown seaweed-derived fucoidan from the Indian Coastline: A natural health product candidate

**DOI:** 10.1371/journal.pone.0346712

**Published:** 2026-04-07

**Authors:** Manisha Prajapati, Dharmendra Kumar, Shivankar Agrawal, Dhiraj Kumar, Pooja Baweja, Dinabandhu Sahoo

**Affiliations:** 1 Department of Botany, University of Delhi, New Delhi, Delhi, India; 2 Department of Botany, Miranda House, University of Delhi, New Delhi, Delhi, India; 3 APC Microbiome Ireland, School of Microbiology, University College Cork, Cork, Ireland; 4 All India Institute of Medical Science, New Delhi, Delhi, India; 5 Department of Botany, Maitreyi College, University of Delhi, New Delhi, Delhi, India; Benha University, EGYPT

## Abstract

Brown seaweeds are rich in bioactive constituents, among which fucoidan a sulphated polysaccharide is particularly noted for its broad-spectrum therapeutic properties. This study explores the multifaceted bioactivity of fucoidan extracted from six brown seaweed species (*Sargassum wightii*, *Sargassum tenerrimum*, *Sargassum cinereum*, *Turbinaria conoides*, *Padina boergesenii*, and *Padina tetrastromatica*) collected from the Pudumadam coast, Gulf of mannar region, Tamil Nadu, India. Fucoidan was isolated and structurally characterized using Fourier-transform infrared spectroscopy (FT-IR) and nuclear magnetic resonance (NMR) spectroscopy. The antioxidant capacity of the extracts was assessed through 2,2-diphenyl-1-picrylhydrazyl (DPPH), hydroxyl radical scavenging activity (HRSA), and ferric reducing antioxidant power (FRAP) assays, with *Sargassum cinereum* showing the highest antioxidant activity (IC₅₀: 0.342 ± 0.01 mg/ml for DPPH; 1.26 ± 0.02 mg/ml for HRSA; 33.17 ± 0.02 mM Fe (II)/g for FRAP). *In vitro* antidiabetic assays demonstrated that fucoidan from *Sargassum wightii* exhibited the strongest inhibition of α-amylase and α-D-glucosidase (IC₅₀: 0.042 ± 0.02 mg/ml). Additionally, fucoidan from *S. wightii* displayed significant cytotoxic activity against MCF-7 human breast cancer cells. These findings underscore the therapeutic promise of brown seaweed-derived fucoidan as a multifunctional agent, supporting its potential application in the formulation of natural health products.

## Introduction

Brown seaweeds represent an abundant and underexplored marine resource along the Indian coastline, offering a rich repository of bioactive compounds with considerable health and commercial potential [1,2]. Among these, fucoidan, a sulfated polysaccharide primarily found in the cell walls of brown algae such as *Fucus*, *Sargassum*, *Laminaria*, and *Turbinaria*, has garnered significant scientific interest due to its broad spectrum of biological activities [3,4]. Structurally, fucoidans are complex heteropolysaccharides composed predominantly of α-L-fucopyranose units, often linked with other sugar monomers such as glucose, galactose, mannose, uronic acids, and sulfate groups features that are closely associated with their bioactivity [5]. Numerous studies have highlighted fucoidan’s antioxidant, antidiabetic, anticancer, anti-inflammatory, antimicrobial, and anticoagulant properties, making it a promising candidate for applications in pharmaceuticals, nutraceuticals, and functional foods [6,7]. These biological activities vary depending on the seaweed species, harvesting conditions, geographic location, and extraction methods [8]. These biological activities vary depending on the seaweed species, harvesting conditions, geographic location, and extraction methods. However, regional studies focusing on the bioactivity of fucoidan from Indian brown seaweeds remain limited, particularly those examining species from the biodiverse Pudumadam coast in the Gulf of Mannar region, Tamil Nadu.

Chronic diseases such as diabetes mellitus and cancer are on the rise globally, with oxidative stress playing a central role in their pathophysiology [9]. Type 2 diabetes, in particular, is associated with elevated postprandial glucose levels due to the action of digestive enzymes such as α-amylase and α-D-glucosidase [10,11]. Synthetic inhibitors of these enzymes are widely used but often have limitations, including undesirable side effects and limited efficacy. Similarly, traditional cancer therapies can result in significant systemic toxicity and poor patient outcomes. These challenges underscore the need for safe, natural bioactive agents capable of modulating oxidative stress and inhibiting disease-related pathways [10,12].

In this context, fucoidans derived from marine brown algae offer a sustainable and effective therapeutic alternative. This study aims to characterize fucoidan extracted from six brown seaweed species (*Sargassum wightii*, *Sargassum tenerrimum*, *Sargassum cinereum*, *Turbinaria conoides*, *Padina boergesenii*, and *Padina tetrastromatica*) collected from the Pudumadam coast, Gulf of mannar region, Tamil Nadu, India. The biological potential of the extracted fucoidans was assessed using multiple in vitro assays, including antioxidant (DPPH, FRAP and HRSA), antidiabetic (α-amylase and α-D-glucosidase inhibition), and anticancer (cytotoxicity against MCF-7 cells) evaluations. The findings contribute to the growing body of evidence supporting marine algal polysaccharides as multifunctional bioactive agents and emphasize the relevance of India’s coastal biodiversity in developing natural health-promoting candidates for chronic disease management.

Furthermore, Principal Component Analysis (PCA) was applied to examine the relationships among the different biological activities and to identify major patterns of variation among the six seaweed species. This multivariate method helped group the species according to their antioxidant, antidiabetic and other biological potentials providing an integrated understanding of their functional diversity. The analysis also offered clearer insights into species-specific bioactivity patterns and supported a better interpretation of the overall experimental results.

## Materials and methods

### Collection of seaweed samples

The samples were collected from Pudumadam coast in Tamil Nadu, India in the month of June. The samples were thoroughly washed several times with seawater followed by freshwater, shade- dried and stored in airtight zipper bags until further use. Species identification was performed using the standard taxonomic book *Common Seaweeds of India* (Sahoo, 2010). The samples were further identified and authenticated by Prof. Dinabandhu Sahoo, University of Delhi, and the herbarium sheets of each specimen were submitted to and deposited in the Herbarium of the Department of Botany, University of Delhi, India, with the following voucher serial numbers: *Sargassum wightii* (DUH/15770/2024), *Sargassum tenerrimum* (DUH/15768/2024), *Sargassum cinereum* (DUH/15771/2024), *Turbinaria conoides* (DUH/15769/2024), *Padina boergesenii* (DUH/15766/2024), and *Padina tetrastromatica* (DUH/15767/2024).

### Extraction of Fucoidan

The extraction was carried out twice following a modified method [13]. A total of 5 g of dried seaweed sample was suspended in 125 mL of 0.5% (w/v) EDTA solution (Ethylenediaminetetraacetic acid disodium salt; purity 98%, SQ grade; Thermo Fisher Scientific Pvt. Ltd., Product No. 18455, SDS version 3.0; Mumbai, Maharashtra, India) in a 1:25 (w/v) ratio. The suspension was continuously stirred in a water bath (Grant Instruments, Model No.- GD120, UK) at 70 °C for 3 h. After incubation, the extract was allowed to cool to room temperature (RT) and filtered using a Büchner funnel (Borosil). Filtrates from both extraction steps were combined and the pH was adjusted to 7.0. To the pooled filtrate, ethanol (purity 99.9%, AR grade; Changshu Yangyuan Chemical Co., Ltd., Model No. XK-13-011-00009; Changshu, Jiangsu, China) was added to reach a final ethanol concentration of 60% (v/v) to precipitate alginic acid. The supernatant was then collected and ethanol was further added until the final concentration reached 80% (v/v) to precipitate fucoidan. The resulting precipitate was centrifuged (Sorvall Legend XFR Centrifuge, Thermo Fisher Scientific, Model No. 75004538, Germany) at 7000 rpm for 10 min, lyophilized and stored at –20 °C for further use. EDTA-based extraction was employed in this study to facilitate the selective recovery of fucoidan while minimizing polysaccharide degradation. EDTA acts as a chelating agent that binds divalent metal ions (e.g., Ca^2+^ and Mg^2+^) involved in cross-linking cell wall components, thereby promoting cell wall disruption and enhancing fucoidan release under mild conditions. This approach avoids the elevated temperatures or acidic conditions commonly associated with hot water or dilute acid extraction, which have been reported to cause depolymerization, desulfation, or structural modification of fucoidan. The use of EDTA for fucoidan extraction has been previously described and justified in the literature. The analytical techniques employed, namely FTIR and NMR spectroscopy, were used exclusively for structural characterization of fucoidan, including confirmation of functional groups, sulfation patterns, and backbone composition, and do not provide molecular weight distribution.

### FT-IR analysis

The fucoidan extracted was analyzed using a Nicolet iS50 FTIR, (Tri-detector, Gold flex, Thermo Fisher Scientific, Massachusetts, USA) spectrophotometer. The analysis was performed in the range of 400–4000 cm ⁻ ¹ in ATR mode to identify characteristic peaks of various functional groups related to fucoidan. The spectral data were analyzed using Origin software for peak interpretation and graphical representation.

### NMR analysis

10 mg of fucoidan was dissolved in 0.5 mL of D₂O (Deuterium oxide; purity 99.9% D, NMR grade, SQ grade, Sigma-Aldrich, St. Louis, MO, USA; part of Merck KGaA, Darmstadt, Germany; Model No. 151882, Version 6.10), ¹H NMR spectra were recorded using a Bruker Avance Neo 400 MHz spectrometer (Standard frequencies: 1 H: 400 MHz, 13 C:100 MHz, temp. facility: −140–180° C, Bruker Corporation Massachusetts, USA). The spectral data were analyzed using MestReNova software.

### Chemical analysis

The fucose content in the extracted fucoidan was determined using the Phenol-sulphuric method [14]. The sulfate content was estimated using the Barium chloride-gelatin method [15]. Uronic acid content was assessed according to the method described by [16]. All experiments were conducted thrice (n = 3).

### Determination of antioxidant capacity

#### DPPH scavenging activity.

The assay was performed in triplicate (n = 3) following the method of [17,18], with minor modifications. A 1.5 mL aliquot of methanolic DPPH solution (0.1 mM; Extrapure, 95% grade; SRL – Sisco Research Laboratories Pvt. Ltd., Product Code 29128, SDS Version 1.4; Taloja, Maharashtra, India) was mixed with 75 µL of fucoidan extract at various concentrations (2.0, 1.0, 0.5, 0.25, and 0.125 mg/mL). The mixtures were incubated at room temperature in the dark for 30 min. A standard calibration curve was prepared using L-ascorbic acid (purity 99%, SQ grade; Thermo Fisher Scientific Pvt. Ltd., Product No. 18455, SDS Version 3.0; Mumbai, Maharashtra, India) at concentrations ranging from 5 to 200 µg/mL. A mixture containing methanol and DPPH solution (without sample) served as the control, while methanol as the blank. Absorbance was measured at 517 nm using a UV–Visible spectrophotometer (Model DU 730, Beckman Coulter Inc., Brea, California, USA). Antioxidant activity was expressed as IC₅₀, defined as the concentration of extract required to inhibit 50% of the initial DPPH radical concentration. IC₅₀ values were calculated from the regression equation obtained by plotting the percentage of inhibition against sample concentration.


$$\%\text{ Inhibition}=({\text{A}}_{\text{0}}-{\text{A}}_{\text{s}}/{\text{A}}_{\text{0}})\text{ 100},$$

where A₀ was the absorbance of the control (DPPH solution without sample) and Aₛ was the absorbance of the sample. The IC₅₀ value was determined from the regression equation plotted between percentage inhibition and sample concentration.

#### ABTS scavenging activity.

This assay evaluates the ability of antioxidants to neutralize the generated ABTS radicals. Equal volumes of 7 mM ABTS (extra pure 98% HPLC grade, SRL-Sisco Research Laboratories Pvt. Ltd., P Code-28042, SDS version 4.0, Taloja, Maharashtra, India) and 2.45 mM Potassium persulphate (extra pure 98% AR grade, SRL-Sisco Research Laboratories Pvt. Ltd., P Code-48319, SDS version 4.0, Taloja, Maharashtra, India) were mixed to prepare the stock solution. The mixture was incubated in the dark for 12 h at room temperature (RT) to generate ABTS^+^ free radicals. Each time the working solution was freshly prepared by diluting the stock with 50% of methanol (purity-99.5% GC grade, Thermo Fisher Scientific- Pvt. Ltd, Product No.-32405, SDS version-2.0, Mumbai, Maharashtra, India) to obtain an absorbance of 0.70 ± 0.02 at 745nm and 30°C. Subsequently, 1200 µL of ABTS working solution was mixed with 120 µL of fucoidan extract at different concentrations (2 mg/ml, 1 mg/ml, 0.5 mg/ml, 0.25 mg/ml, 0.125 mg/ml), followed by incubation for 6 min at 30°C at 745 nm. The absorbance was recorded at 745 nm against methanol as the blank, using quercetin as the standard. The results were expressed as mean ± standard deviation (SD) from three independent replicates (n = 3) and the IC_50_ values were calculated as described earlier [19].

#### HRSA scavenging activity.

The activity was tested as per [20]. A 500 µl extract of different concentration (2 mg/ml, 1 mg/ml, 0.5 mg/ml, 0.25 mg/ml, 0.125 mg/ml) was taken and 500 µl of 1.5mM Ferrous sulfate (purity-98% SQ grade, Thermo Fisher Scientific Pvt. Ltd, Product No.-23755, SDS version-2.0, Mumbai, Maharashtra, India), 350 µl of 6 mM H_2_O_2_ (50%, EMPLURA®, Merck Life Sciences Pvt. Ltd, Material code 1.93408.0521, Mumbai, Maharashtra, India) and 150 µl of Sodium salicylate (purity-99.5% SQ grade, Thermo Fisher Scientific Pvt. Ltd, Product No.-28035, Mumbai, Maharashtra, India),) were added. The mixture was incubated for 1 hour at RT. The final volume was adjusted to 5 ml using distilled water and the absorbance was measured at 510 nm using a UV–Visible spectrophotometer (Model DU 730, Beckman Coulter Inc., Brea, California, USA). A calibration curve was prepared using ascorbic acid as the standard. Each assay was conducted in triplicate (n = 3) to ensure reproducibility. Scavenging activity IC_50_ calculated using;


$$\text{OH}-\text{radical scavenging }\%=\text{1}-({\text{A}}_{\text{1}}-{\text{A}}_{\text{2}})/{\text{A}}_{\text{0}}*\text{100 }$$

where, A_0_ = control without sample, A_1_ = sample, A_2_ sample without sodium salicylate.

#### Nitic Oxide scavenging activity.

Nitic oxide activity was assessed according to [21] with minor modifications. A 10 mM sodium nitroprusside solution in phosphate-buffer saline (PBS) (molecular biology grade, SRL-Sisco Research Laboratories Pvt. Ltd., P Code-78529, SDS version 2.0, Taloja, Maharashtra, India) was used as the reaction mixture. 2 ml of the reaction mixture was mixed with 200 µl fucoidan extract of different concentrations. The mixture was incubated at 37°C for 1hr. Then, 500 µl of Griess reagent (extra pure 98%, SRL-Sisco Research Laboratories Pvt. Ltd., P Code-28042, SDS version 4.0, Taloja, Maharashtra, India) was added. The percent inhibition was calculated by taking absorbance at 540 nm and IC_50_ values were determined as described before. Measurements were taken in triplicate (n = 3) and results were expressed as mean ± standard deviation. PBS was used as the blank and BHA (Butylated hydroxyanisole) (purity 98% GC grade, SRL-Sisco Research Laboratories Pvt. Ltd., P Code-60004, SDS version 4.0, Taloja, Maharashtra, India) was taken as positive control.

#### FRAP assay.

The assay was carried out according to [22]. Fresh FRAP reagent was prepared by mixing 300 mM sodium acetate anhydrous (purity 98% SQ grade, Thermo Fisher Scientific India Pvt. Ltd. P Code- 20125, Mumbai, Maharashtra, India) buffer (pH 3.6), TPTZ (2,4,6-tri (2-pyridyl)-s-triazine) solution (extra pure 99% AR grade, SRL-Sisco Research Laboratories Pvt. Ltd., P Code-25793, SDS version 4.0, Taloja, Maharashtra, India) and 20 mM FeCl_3_ anhydrous (pure 98%, SRL-Sisco Research Laboratories Pvt. Ltd., P Code-72287, Taloja, Maharashtra, India) in a 10:1:1 ratio. The mixture was incubated in dark for 10 minutes. Then, 60 µl of fucoidan extract, 90 µl of sodium acetate buffer and 1.5 ml of FRAP reagent were mixed, followed by incubation in dark for 30 minutes. The analysis was performed in triplicates (n = 3) to ensure consistency of results. A calibration curve was prepared using ferrous sulfate (FeSO₄.7H₂O) as the standard. Absorbance was measured at 593 nm, using acetate buffer as blank. The antioxidant capacity was determined using the regression equation y = 0.464x − 0.0114 (R^2^ = 0.9996) where x is the absorbance at 593 nm and y is the concentration varied from 0.1 - 2mM and Results were expressed as mM Fe(II)/g of extract.

#### Inhibition assay for α-D-glucosidase enzyme activity.

Assay was determined in independent triplicates (n = 3) as per the modified method of [23,24]. Different fucoidan concentrations (320 µl aliquots each) were mixed with 60 µl of α-D-glucosidase enzyme solution (ex. Yeast, SRL-Sisco Research Laboratories Pvt. Ltd., P Code- 75551, SDS version 1.0, Taloja, Maharashtra, India) and 500 µl of Sodium Phosphate Buffer (SPB). Incubation was carried out at 37°C for 15 min in a water bath to allow enzyme-fucoidan interaction. Then, 320 µl of 2 mM PNP-G (p-Nitrophenyl-β-D-Galactopyranoside) (extra pure 98%, SRL-Sisco Research Laboratories Pvt. Ltd., P Code-12735, Taloja, Maharashtra, India) was added after incubation, and the mixture was incubated again for 10 min at 37°C. Subsequently, 500µl of sodium carbonate (NaCO_3_) (purity-99.5% SQ grade, Thermo Fisher Scientific Pvt. Ltd, Product No.-27555, SDS version-2.0, Mumbai, Maharashtra, India) was added to stop the reaction. Absorbance was measured at 405 nm using a UV–Visible spectrophotometer (Model DU 730, Beckman Coulter Inc., Brea, California, USA). A blank was prepared for each fucoidan concentration by replacing both enzyme and substrate with SPB. A control was prepared by replacing enzyme solution with SPB. Acarbose was used as the reference standard to compare the inhibitory activity.

#### Inhibition assay for α-amylase activity.

The α-amylase inhibitory activity was determined according to the modified method [18,23,24]. Briefly, 200 µL of fucoidan extract was mixed with 200 µL of α-amylase enzyme solution (ex. Porcine pancreas; SRL–Sisco Research Laboratories Pvt. Ltd., Product Code 28588, SDS version 1.0; Taloja, Maharashtra, India). The reaction mixture was incubated at 37 °C for 15 min. After incubation, 600 µL of starch solution was added to each tube as a substrate and the mixtures were further incubated for 5 min. The reaction was then terminated by adding 1 mL of 3,5-dinitrosalicylic acid (DNSA) reagent (purity 97%, synthesis grade; Loba Chemie Pvt. Ltd., Product No. 03480, SDS version 1.0; Mumbai, Maharashtra, India). The tubes were incubated in a boiling water bath at 100 °C to allow colour development followed by cooling to room temperature. The absorbance was measured at 540 nm using a UV–visible spectrophotometer (Model DU 730, Beckman Coulter Inc., Brea, California, USA). All analyses were conducted in triplicate. Appropriate blank and control samples were prepared under identical conditions to ensure accuracy. Acarbose served as the standard reference.

#### Cell viability assay (MTT detection assay).

The *in vitro* cytotoxicity of fucoidan was determined against MCF-7 cancer cells by MTT assay (3-(4,5-dimethylthiazol-2-yl)-2,5-diphenyl-2H-tetrazolium bromide, purity 99.5% TLC grade, Sigma-Aldrich, Merck KGaA, USA, P Code: M2003) according to [25]. Cells were seeded in triplicates in 96-well plates and cultured. Ten microliters (10 µl) of fucoidan (2 mg/ml) were added to each well and incubated for 48 h. Afterwards, 10 µl of MTT reagent (Invitrogen) was added per 100 µl of medium to each well, and incubated for 4 h at 37°C. The resulting formazan crystals were then dissolved in 50 µl of dimethyl sulfoxide (DMSO) (99%, EMPLURA®, Merck Life Sciences Pvt. Ltd, P code: 1.16743.0521, Mumbai, Maharashtra, India) and incubated at 37°C for 15 min. The optical density was measured using a microplate reader (Bio-Rad Laboratories, Inc., Model 680, USA) at 540 nm. All analyses were conducted in triplicate (n = 3). Doxorubicin was used as the standard.


$$\%\text{ Viability }=\;({\text{OD}}_{\text{treated sample}}-{\text{OD}}_{\text{blank}})\;/\;({\text{OD}}_{\text{control }}-{\text{OD}}_{\text{blank}})*\text{100}$$

### Statistical analysis

The statistical one-way single factor analysis of variance (ANOVA) followed by Duncan’s post hoc comparison test for the data set was performed to compare the means of different treatments at p < 0.05. Each experiment was conducted in triplicate (n = 3), and the results were expressed as mean ± standard deviation. The coefficient of variation (CV%) was calculated to assess the relative variability of the observed data. CV% expresses the standard deviation as a percentage of the mean and allows comparison of variability among datasets with different units or scales. Multivariate Principal Component Analysis (PCA) was carried out using OriginPro 9.0 software and Agglomerative hierarchical Clustering (AHC), Score plot, Biplot and Box plot were generated. PCA was applied to reduce data dimensionality and identify the principal components contributing to variance among the biological activities. This analysis enabled visualization of correlations between antioxidant, antidiabetic and other parameters allowing clear differentiation among seaweed species based on their bioactivity profiles and facilitating the interpretation of complex, multidimensional data.

## Results

### Yield

Fucoidan yield varied notably among species with the highest in *Sargassum tenerrimum* (8.4 ± 0.047%) and *S. wightii* (8 ± 0.094%), and the lowest in *Turbinaria conoides* (4.4 ± 0.081%). Higher yields in *Sargassum* species likely reflect their greater polysaccharide and sulfate content, enhancing extraction efficiency. Comparable yields (6–9%) were previously reported for *Sargassum* spp. [26,27]. In contrast, lower yields in *Padina* and *Turbinaria* may stem from structural differences and reduced fucoidan abundance.

The fucose/sulphate ratio of fucoidan varied among the examined brown seaweed species, ranging from 3.46 to 6.85. *Sargassum cinereum* (3.46) and *S. wightii* (3.92) showed relatively lower ratios, whereas *Padina tetrastromatica* exhibited the highest value (6.85), followed by *S. tenerrimum* (5.83). A lower fucose/sulphate ratio generally indicates a higher degree of sulphation which enhances the bioactivity of fucoidan, particularly its antioxidant, anticoagulant and anticancer properties [28]. In contrast, higher ratios suggest the presence of more neutral sugars or carbohydrate impurities, reflecting less sulphated and possibly less purified fractions [29].Therefore, the lower ratios observed in *S. cinereum* and *S. wightii* suggest better purification and enrichment of sulphated fucoidan, whereas the higher ratios in *Padina* species indicate a comparatively lower sulphate substitution or partial co-extraction of neutral polysaccharides such as alginate or laminaran. Although sulphation is often considered a key contributor to fucoidan bioactivity, our results reveal several clear exceptions that indicate a more complex, multifactorial relationship. *S. wightii*, despite exhibiting a high degree of sulphation, showed the lowest FRAP antioxidant activity, suggesting that FRAP-based antioxidant capacity is not solely governed by sulphate content but is also influenced by factors such as molecular weight, monosaccharide composition, glycosidic linkages, and overall molecular conformation. Similarly, *T. conoides*, which possessed the highest sulphation level among the samples, demonstrated only moderate anticancer activity, indicating that anticancer effects depend on additional structural parameters, including sulphate positioning and branching patterns. Furthermore, fucoidans with comparable fucose/sulphate ratios displayed contrasting enzyme inhibition profiles: *S. wightii* (ratio 3.92) showed strong α-amylase but moderate α-D-glucosidase inhibition, whereas *S. cinereum* (ratio 3.46) exhibited the opposite trend, highlighting enzyme-specific selectivity likely arising from subtle structural variations. Collectively, these observations demonstrate that similar sulphation profiles can lead to divergent biological activities. Accordingly, the original hypothesis has been revised and qualified to emphasize that sulphation is an important, but not exclusive, determinant of bioactivity, and that synergistic effects of multiple structural features govern the functional outcomes of fucoidan.

### FT-IR analysis

**Fig 1:**
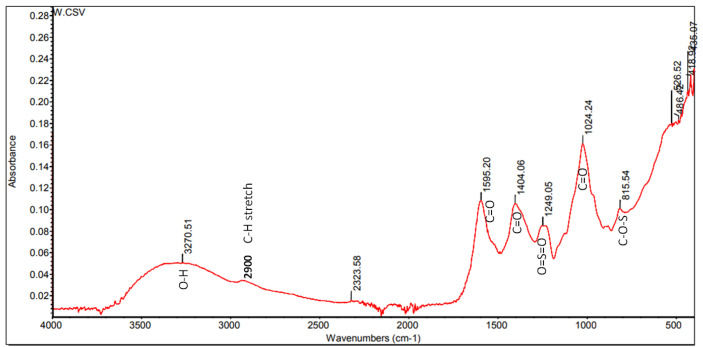
FT-IR Spectra of crude Fucoidan from *Sargassum wightii.*

The FT-IR spectra of fucoidan were depicted in **Fig 1**. **Table 1** showing observed peaks of FT-IR analysis of Fucoidan and comparison with standard Fucoidan [30–32] The band at approximately 1024 cm^-1^ corresponds to the C-O vibrations of the polysaccharides. The absorption around 1249 cm^-1^ indicates the O = S = O stretching of sulfates, serving as a marker for sulfated polysaccharides. The absorption at 1595 cm^-1^ corresponds to carbonyl (C = O) groups of uronic acid, while the stretching at 1404 cm^-1^ corresponds to C-O within the –COOH group. The band around 3270 cm^-1^ corresponds to O-H stretching, a common peak for sugars. The C-H stretching band at approximately 2900 cm^-1^ corresponds to the methyl groups of fucose. The band at approximately 815 cm^-1^ corresponds to C-O-S bending vibrations of sulfate groups, primarily located at the C-2, C-3 or C-4 position.

**Table 1:** Table showing observed peaks of FT-IR analysis of Fucoidan and comparison with Standard Fucoidan (from literature).

Observed Peak (cm ⁻ ¹)	Functional Group	Standard Fucoidan Peak (cm ⁻ ¹)	Remarks
**3270.51**	O–H stretching (hydroxyl groups)	3400–3200	Broad band typical of polysaccharides
**2900**	C–H stretching	2920–2930	Matches aliphatic CH stretching region
**2323.58**	CO₂ overtone or atmospheric interference	—	Not characteristic of fucoidan
**1595.20**	C = O stretching (uronic acid or amide)	1600–1650	Indicates uronic acid residues or amide impurities
**1404.06**	C–O symmetric stretch/ COO⁻	1400–1420	Carboxylate or uronic acid vibration
**1249.05**	Asymmetric S = O stretching (sulfate ester)	1250–1240	Strong indicator of sulfation in fucoidan
**1024.24**	C–O–C and C–O stretching	1030–1020	Polysaccharide backbone vibrations
**815.54**	C–O–S stretching vibration	820–850	Characteristic sulfate group (axial sulfate)
**486.52/ 436.07**	Fingerprint region	—	Non-diagnostic carbohydrate region features

### NMR analysis

**Fig 2:**
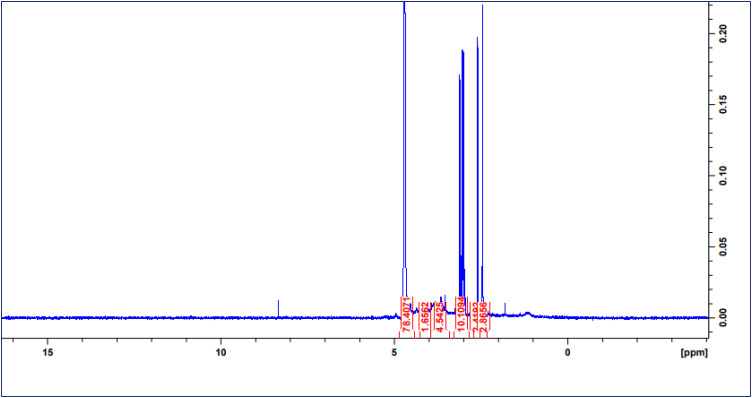
^1^H NMR analysis of crude fucoidan from *Sargassum wightii.*

The ^1^H NMR spectrum of fucoidan shown in **Fig 2**. The signal at 4.54 ppm corresponds to 3-linked α-L-fucose, a major fucoidan component while the signal at 1.6 ppm represents the methyl protons of fucopyranose residues [33]. The signal at 1.2 ppm indicates the presence of alkane protons in methyl groups. The signal at 2.4 ppm corresponds to protons attached to alkyl groups linked to sulfonyl moieties whereas the signals between 3–4 ppm corresponds to methoxy-linked proton. The signal at 3.5 ppm is attributed to α-L rhamnosyl residues.

### Determination of Fucose content in Fucoidan

**Fig 3:**
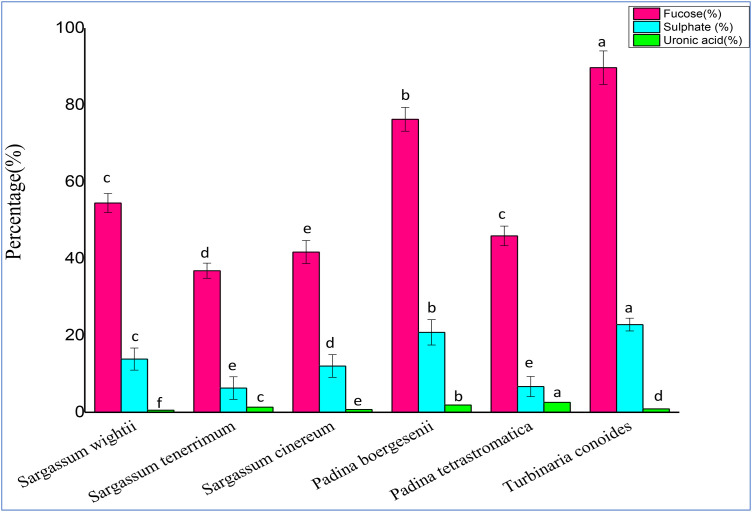
Fucose, Sulphate and Uronic acid content of fucoidan. Values are presented as Mean ± SD (n = 3). Different superscript letters (a, b, c, d, f and e) indicate significant differences at p < 0.05 by one-way ANOVA followed by Duncan’s post hoc comparison test.

Fucose content (% dry weight) was analyzed among six different species of brown seaweeds and was found to vary from 36.85–89.68%. The lowest fucose sugar content (36.85% ± 0.02) was observed in *Sargassum tenerrimum,* followed by 41.71% ± 0.01 in *Sargassum cinereum* while the highest content was recorded in *Turbinaria conoides* with 89.68% ± 0.01 (**Fig 3**).

### Determination of Sulfate content

Sulfate content was analyzed among six different brown seaweed species. It varied from 6–20%. The highest sulfate content was observed in *Turbinaria conoides* with 22.83% ± 0.016 *Sargassum cinereum* and *Sargassum wightii* showed sulfate contents of 12.05% ± 0.01 and 13.85% ± 0.01 respectively. *Sargassum tenerrimum* had lowest sulfate content of 6.31% ± 0.01 as shown in **Fig 3**.

### Determination of Uronic acid content

**Fig 3** shows the uronic acid content analyzed in six brown seaweed species which from 0.5–3%. The highest uronic acid content was observed in *Padina tetrastromatica* with 2.6% ± 0.01 while *Sargassum wightii* showed the lowest content of 0.55% ± 0.01.

### Determination of antioxidant capacity

#### DPPH scavenging activity.

**Fig 4:**
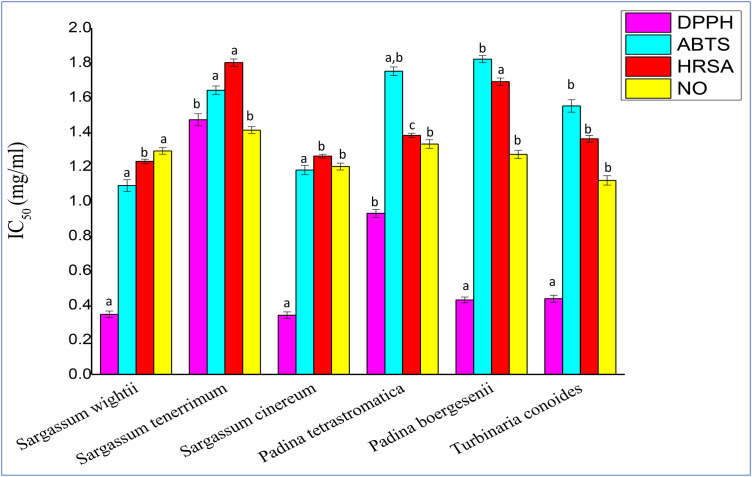
Antioxidant activity (IC_50_) of crude fucoidan through DPPH, ABTS, HRSA, NO. Values are presented as Mean ± SD (n = 3). Different superscript letters (a, b and c) indicate significant differences at p < 0.05 by one-way ANOVA followed by Duncan’s post hoc comparison test.

DPPH scavenging activity of six fucoidan samples from brown seaweeds is shown in **Fig 4**. It was observed that *Sargassum cinereum* and S*argassum wightii* exhibited the highest DPPH activity with IC_50_ of 0.342 ± 0.02 mg/ml and 0.347 ± 0.012 mg/ml respectively, while *Sargassum tenerrimum* 1.47 mg/ml ± 0.035 showed the lowest activity.

#### ABTS scavenging activity.

ABTS scavenging activity of six fucoidan samples is depicted in **Fig 4**. *Sargassum wightii* (IC_50_ 1.09 ± 0.034 mg/ml) exhibited the highest activity followed by *Sargassum cinereum* (IC_50_ 1.18 ± 0.027 mg/ml), while *Padina boergesenii* (IC_50_ 1.82 ± 0.021 mg/ml) showed the lowest radical eliminating capacity.

#### HRSA scavenging activity.

**Fig 4** shows the scavenging activities of six fucoidan samples against the hydroxyl radicals. The highest activity was observed in *Sargassum wightii*; (IC_50_ of 1.23 ± 0.012 mg/ml) and in *Sargassum cinereum* (IC_50_ 1.26 ± 0.01 mg/ml). *Padina boergesenii* (IC_50_ 1.69 ± 0.021 mg/ml) and *Sargassum tenerrimum* (IC_50_ 1.80 ± 0.021 mg/ml) showed the lowest activity.

#### Nitric Oxide scavenging activity.

Nitric Oxide assay of fucoidan samples is shown in Fig 4. *Turbinaria conoides* (IC_50_ 1.12 ± 0.018 mg/ml) exhibited the highest activity, followed by *Sargassum cinereum* (IC_50_ 1.20 ± 0.022 mg/ml) and *Sargassum wightii* (IC_50_ 1.29 ± 0.013 mg/ml), while *Padina tetrastromatica* (IC_50_ 1.33 ± 0.013 mg/ml) showed the least activity.

#### FRAP assay.

**Fig 5:**
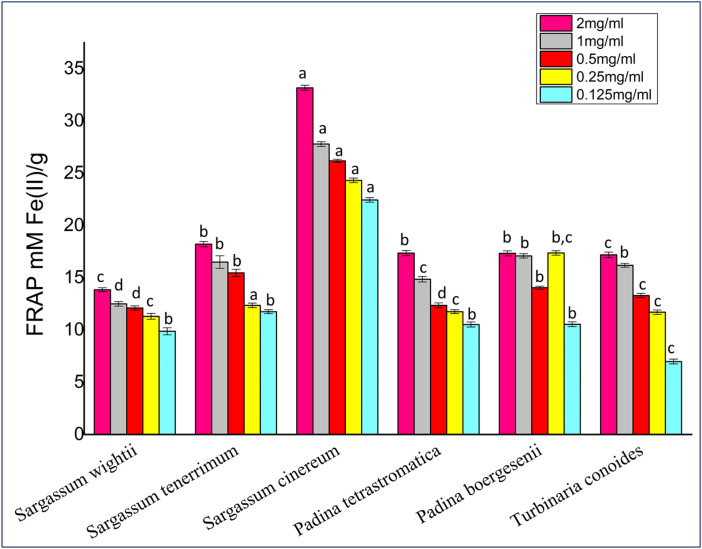
FRAP activity of crude fucoidan. Values are presented as Mean ± SD (n = 3). Different superscript letters (a, b, c and d) indicate significant differences at p < 0.05 by one-way ANOVA followed by Duncan’s post hoc comparison test.

FRAP activity of all fucoidan samples is depicted in Fig 5. *Sargassum cinereum* (33.17 ± 0.02mM Fe (II)/g extract) showed the highest FRAP activity, followed by *Sargassum tenerrimum* (18.21 ± 0.02 Fe (II)/g extract), *Padina tetrastromatica* (17.36 ± 0.025 Fe (II)/g extract), *Padina boergesenii* (17.37 ± 0.024 Fe (II)/g extract) and *Turbinaria conoides* (17.18 ± 0.027 Fe (II)/g extract), while the lowest activity was observed in *Sargassum wightii* (13.84 ± 0.19 mM Fe (II)/g extract).

### Enzymatic activity

#### α- amylase inhibitory activity.

**Fig 6:**
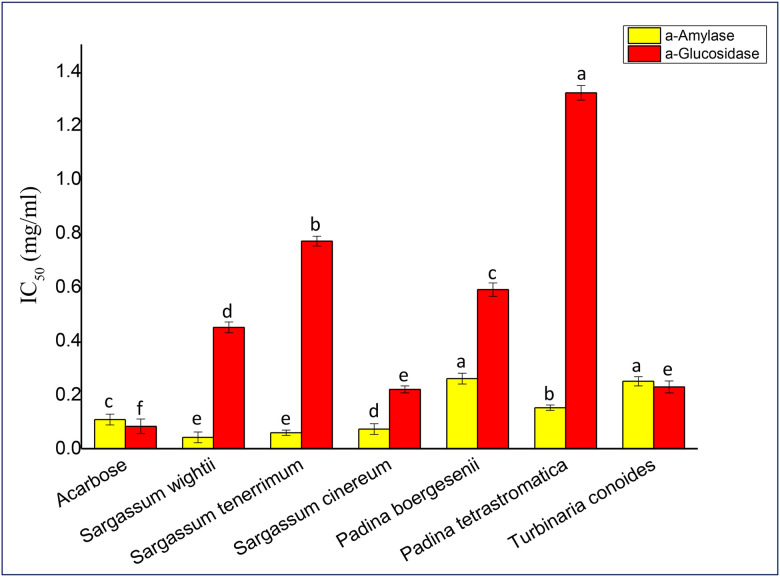
Enzymatic activity of crude fucoidan against α- amylase and α- glucosidase. Values are presented as Mean ± SD (n = 3). Different superscript letters (a, b, c, d, e and f) indicate significant differences at p < 0.05 by one-way ANOVA followed by Duncan’s post hoc comparison test.

The inhibitory potential of fucoidan against α- amylase and α-D-glucosidase using PNP-G as the substrate was screened. Inhibitory activity of fucoidan from all six seaweeds against α- amylase is shown in Fig 6. The highest activity was found in *Sargassum wightii* having IC_50_ 0.042 ± 0.02 mg/ml making it the most effective, followed by *Sargassum tenerrimum* (IC_50_ = 0.059 ± 0.01 mg/ml) and, *Sargassum cinereum* (IC_50_ = 0.0728 ± 0.02 mg/ml). *Padina tetrastromatica* has (IC_50_ = 1.152 ± 0.01 mg/ml) and was the least effective.

#### α-D-glucosidase inhibitory activity.

Inhibitory activity of fucoidan from all six seaweeds against α-D-glucosidase is shown in Fig 6. *Sargassum cinereum* was found to be the most effective (IC_50_ = 0.22 ± 0.013 mg/ml), followed by *Turbinaria conoides* (IC_50_ = 0.229 ± 0.022 mg/ml), *Sargassum wightii* (IC_50_ 0.45 ± 0.02 mg/ml) while *Padina tetrastromatica* (IC_50_ = 1.32 ± 0.19 mg/ml) showed the least effectiveness.

### Cytotoxicity of fucoidan on MCF7 cancerous cells

**Fig 7:**
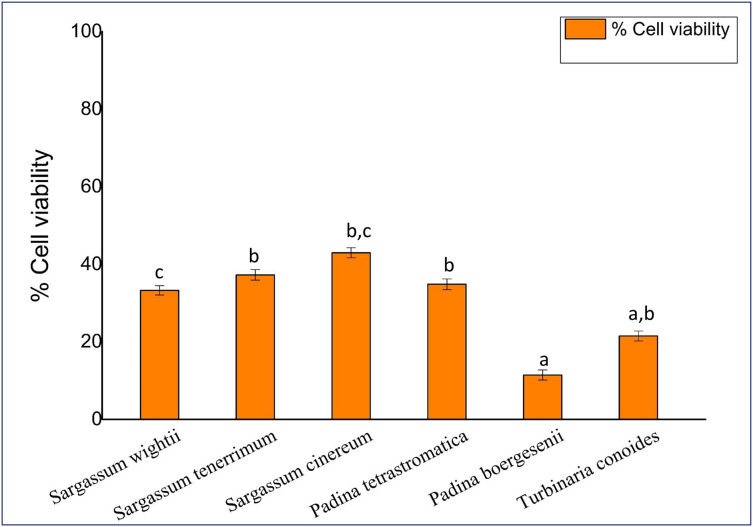
Cytotoxic effect of crude fucoidan. Values are presented as Mean ± SD (n = 3). Different superscript letters (a, b and c) indicate significant differences at p < 0.05 by one-way ANOVA followed by Duncan’s post hoc comparison test.

The anticancer activity of fucoidan (at 2 mg/mL) on MCF-7 breast cancer cell lines is depicted in Fig 7. In this study, among the species; cells treated with fucoidan extracted from *Padina boergesenii* exhibited the highest toxicity, with only 11.43% ± 0.044 cell viability. This was followed by *Turbinaria conoides* (21.5% ± 0.143), *Sargassum wightii* (33.26% ± 0.103), *Padina tetrastromatica* (34.84% ± 0.174) and *Sargassum tenerrimum* (37.27% ± 0.119). The least toxicity was observed in *Sargassum cinereum*, which showed 42.96% ± 0.199 viability. Viable cells actively convert MTT into the colored compound formazan. The results were statistically significant (p < 0.05) when compared with other samples.

### Statistical analysis

Results were expressed as the mean of the replicates ± standard deviation. One-way single factor ANOVA was performed on the dataset to analyze various antioxidant; enzymatic and chemical activities compare the significant differences in the means of different treatments at p < 0.05. Results are shown in Table 2. It was found to be highly significant with p-value of 8.44E-11, 0.038711 and 3.41E-08 which were much lower than 0.05; a similar result was found for the F-value, where F > F_critical_. The coefficient of variation (CV%) values indicated acceptable experimental precision across all parameters, with relatively low variability observed for fucose sugar, sulphate content, uronic acid, antioxidant, and enzyme inhibitory activities among the studied seaweed species. Overall, *Turbinaria conoides* and Padina spp. showed more consistent results (lower CV%), while comparatively higher variability was observed in α-amylase inhibition and cell viability across species (Table 3). The higher CV% values (>60%) observed in some species may be attributed to the inherent variability of the MTT assay, including its sensitivity to differences in metabolic activity and cell seeding density, as well as species-specific cellular responses.

**Table 2:** (A) Single factor ANOVA analysis of dataset for antioxidant activities. (B) Single factor ANOVA analysis of dataset for enzymatic activities. (C) Single factor ANOVA analysis of dataset for chemical activities.

A. Single Factor ANOVA analysis of dataset for Antioxidant activities						
ANOVA						
*Source of Variation*	*SS*	*df*	*MS*	*F*	*P-value*	*F crit*
Between Groups	1610.136	4	402.534	42.48262	8.44E-11	2.75871
Within Groups	236.8816	25	9.475263			
Total	1847.017	29				
**B. Single Factor ANOVA analysis of dataset for Enzymatic activities**						
**ANOVA**						
***Source of Variation***	***SS***	***df***	***MS***	***F***	***P-value***	***F crit***
Between Groups	0.201554	1	0.201554	5.657282	0.038711	4.964603
Within Groups	0.356273	10	0.035627			
Total	0.557827	11				
**C. Single Factor ANOVA analysis of dataset for Chemical activities**						
**ANOVA**						
***Source of Variation***	***SS***	***df***	***MS***	***F***	***P-value***	***F crit***
Between Groups	13019.49	3	4339.83	35.41918	3.41E-08	3.098391
Within Groups	2450.554	20	122.5277			
Total	15470.04	23				

**Table 3:** Coefficient of variation (CV%) for monosaccharide fucose sugar, sulphate content, uronic acid, sulphate/fucose ratio, yield, antioxidant activities (DPPH, ABTS, HRSA, NO, and FRAP assays), enzyme inhibitory activities (α-amylase and α-glucosidase IC₅₀), and cell viability (%) of crude fucoidan extracted from different brown seaweed species.

S.NO.	CV (%)	CV (%) of Fucose sugar	CV (%) of Sulphate content	CV (%) of Uronic acid	CV (%) of Yield	CV(%) of DPPH IC50 (mg/ml)	S.NO.	CV (%)	CV (%) of Fucose sugar	CV (%) of Sulphate content	CV (%) of Uronic acid	CV (%) of Yield	CV(%) of DPPH IC50 (mg/ml)	S.NO.
1.	Sargassum wightii	2.584	1.777	7.362	1.168	6.828	7.869	3.721	4.410	4.9674	5.225	13.961	33.26 ± 0.103	37.50
2.	*Sargassum tenerrimum*	10.835	5.029	5.031	0.556	7.284	6.964	5.215	4.126	4.416	4.313	7.049	37.27 ± 0.119	40.56
3.	*Sargassum cinereum*	4.902	2.432	15.062	0.679	7.023	6.551	5.099	4.168	2.269	5.150	8.777	42.96 ± 0.199	62.63
4.	*Padina boergesenii*	3.884	1.563	3.268	0.790	6.510	5.958	4.411	3.524	5.392	5.190	6.900	34.84 ± 0.174	61.68
5.	*Padina tetrastromatica*	4.530	3.598	1.420	0.657	7.174	5.248	5.278	3.749	5.063	4.392	7.352	11.43 ± 0.044	24.68
6.	*Turbinaria conoides*	2.355	1.82	4.641	1.855	8.677	6.150	4.744	4.401	5.796	6.047	7.854	21.5 ± 0.143	64.07

## Discussion

Brown seaweeds represent a valuable source of bioactive compounds with wide-ranging pharmacological and nutraceutical potential. Among these, fucoidan a complex sulfated polysaccharide is emerging biological and therapeutic relevance due to its diverse biological activities. Fucoidan exhibits potent antioxidant, anti-inflammatory, antiviral, anticoagulant and anticancer properties with its antioxidant capacity being particularly important for neutralizing free radicals and mitigating oxidative stress. Beyond its antioxidant function, it also modulates immune responses, promotes wound healing and supports cardiovascular health, underscoring its value in pharmaceutical and cosmeceutical formulations. The bioactivity of fucoidan however, varies considerably among species and is largely influenced by factors such as algal source, structural complexity, degree of sulfation, acetylation, methylation, monosaccharide composition, uronic acid content and extraction technique. Therefore, optimizing extraction parameters is crucial to obtain fucoidan with consistent purity and biological efficacy. In the present study, EDTA-based extraction proved effective yielding fucoidan fractions devoid of detectable polyphenols or EDTA residues. This high degree of purity ensures the reliability of subsequent structural and biological evaluations, thereby establishing a strong foundation for assessing the therapeutic potential of fucoidan derived from Indian brown seaweeds.

FT-IR analysis revealed functional groups characteristic of fucoidan, aligning to a significant degree, with those of earlier conducted research works by [34] and [20]. Absorption bands between 2853–2989 cm ⁻ ¹ indicated CH stretching of the pyranose ring and the C6 group in fucose and galactose. S = O groups, identified in the spectra of *P. pavonica* (1195–1237 cm ⁻ ¹) and S. vulgare (1210–1280 cm ⁻ ¹), are characteristic of fucoidan [35]. The ¹H NMR spectrum displayed peaks at 3.92 ppm (attributable to β-d-galactose) and 3.5–4.5 ppm (ring protons indicating sulfate groups), consistent with the study [34].

The fucose content of fucoidan showed marked variation among the examined brown seaweeds, reflecting clear species-specific differences in polysaccharide composition. It was further influenced by extraction method, seasonal variation and collection site. *Sargassum tenerrimum* showed the lowest fucose proportion (36.85% ± 0.02), whereas *Turbinaria conoides* contained the highest level (89.68% ± 0.01). These findings are consistent with earlier studies reporting 39.04% in *S. tenerrimum* [36], 59.1% in *S. illicifolium* [37], 53% in *Sargassum wightii* as reported [38]. The fucose content in *T. conoides* was notably higher than that in *T. decurrens* (59.3%) [39], highlighting interspecific variation and further support that *Turbinaria* species serve as richer sources of fucose-based fucoidan. Since higher fucose content has been linked to greater antioxidant, anticoagulant activities, given fucose’s crucial role in the structural integrity and bio functionality of fucoidans. Conversely, the comparatively lower fucose content in *Sargassum* species may indicate a more heterogeneous polysaccharide composition, potentially influencing their overall bioactivity. However, when comparing species-specific bioactivities, it is important to consider that variations in molecular weight (MW) may also contribute to the observed differences, as MW is a key determinant of fucoidan functionality. Low sugar levels are preferred in functional foods for their low glycemic impact [16].

Sulfate content and its positional distribution are key determinants of fucoidan bioactivity [29]. They enhance the molecule’s negative charge density, facilitating interactions with enzymes such as α-amylase which can inhibit enzyme activity, suppress cell proliferation and contribute to antioxidant properties [30]. In the present study, sulfate levels varied notably among species, ranging from 6.31 ± 0.01% in *Sargassum tenerrimum* to 22.83 ± 0.016% in *Turbinaria conoides*. It corroborates previous finding including *Undaria pinnatifida* (22.83 ± 1%) [15], *Laminaria japonica* (27.95%) [12] *Padina boergesenii* (17.72 ± 10.25%) [28] and *S. fluitans* (7.56%) [33].

Uronic acid in fucoidan contributes significantly to its anticoagulant potential by interacting with plasma proteins and modulating blood lipid profiles [34]. In the present study, uronic acid levels were relatively low, with *Padina boergesenii* containing 1.91 ± 0.015% (w/w) and *Sargassum tenerrimum* 1.35 ± 0.016% (w/w). These values align with previous findings for *Undaria pinnatifida* (4.06 ± 0.44%) [15], *Turbinaria ornata* (7.8%) [31] and *Dictyota dichotoma* (2.25 ± 0.80%) [35]. The observed variation suggests that although it is present in smaller amounts compared to fucose or sulfate, uronic acids play a vital structural role, contributing carboxyl groups that enhance the electrostatic interactions of fucoidan with biomolecules involved in coagulation pathways.

The available DPPH‐radical scavenging data show significant differences among these seaweeds. Notably, *Sargassum cinereum* was by far the most potent DPPH scavenger: its (IC_50_ = 0.342 ± 0.019 mg/ml) followed by *S. wightii*. This implies that *S. cinereum*’s polysaccharides are much more effective at donating hydrogen to neutralize DPPH. Such enhanced activity often correlates with a higher sulfate‐to‐fucose ratio in the fucoidan, since sulfate groups strongly boost radical‐scavenging potential. Previous studies show lower activity in *S. binderi* (IC_50_ = 2.01 ± 0.29 mg/ml) [40], *S. hystrix* (IC_50_ = 2200.23 ± 1014.77 ppm) [41] likewise, *L. japonica* has only moderate activity [42]. Other studies include 72.59 ± 0.32% inhibition for *S. wightii* [43] and 55.62% for *P. boergesenii* at 1 mg/ml [44]. The differences likely arise from their distinct polysaccharide and other content as well as possibly differing secondary metabolites.

ABTS scavenging activity among the tested species ranged between 1.09–1.82 mg/ml, with *Sargassum wightii* showing a strong IC₅₀ = 1.09 ± 0.034 mg/ml. The markedly higher activity of *S. wightii* suggests a richer pool of antioxidant constituents, possibly due to a higher phenolic or sulfate content contributing to enhanced radical-quenching ability. However, similar reports found in studies done in *Padina tetrastromatica* (IC₅₀ = 0.5 ± 0.04 mg/ml) and *Turbinaria conoides* (IC₅₀ = 0.1 ± 0.03 mg/ml) [40]. Environmental factors, seasonal changes and extraction conditions may also account for the observed variability in ABTS radical scavenging efficiency among brown seaweeds.

Hydroxyl radical scavenging plays a crucial role in protecting cellular components against oxidative stress-induced damage [41]. In the present study, the observed activity was comparable to that reported for *Sargassum hystrix* (IC₅₀ = 2360.07 ± 536.93 ppm) [32], but lower than that of *S. ilicifolium*, which exhibited an IC₅₀ of 209.99 ± 0.25 µg/ml [25]. Comparatively, *Dictyota* species showed about 8% inhibition at 0.5 mg/ml [45]. while *Undaria pinnatifida* displayed stronger inhibition (76.97 ± 1.69% at 2 mg/ml) [14]. This indicates interspecific variability in hydroxyl radical scavenging efficiency within species. Such differences are likely influenced by variations in structural polysaccharides, degree of sulfation, phenolic composition, environmental and extraction-related factors that modulate the availability of hydrogen-donating compounds.

Nitric oxide (NO) scavenging activity was most pronounced in *P. boergesenii* (IC₅₀ = 1.27 ± 0.023 mg/mL). These findings agree with earlier reports for the same species. (IC₅₀ = 5061.88 µg/mL) [28], *S. polycystum* (IC₅₀ = 5.36 ± 0.08 mg/mL) [39]. This enhanced activity may be attributed to variations in extraction method, bioactive composition or seasonal factors influencing metabolite yield.

Ferric reducing antioxidant power (FRAP), reflecting the electron-donating ability of the extracts, ranged from 17–33 mM Fe (II)/g. *S. cinereum* exhibited the highest reducing capacity (33.17 ± 0.02 mM Fe (II)/g), indicating strong reductive potential compared to *P. tetrastromatica* (IC₅₀ = 135 ± 0.1 mg/mL) and *T. conoides* (IC₅₀ = 241 ± 0.1 mg/mL) [40]. These results align with previous reports such as 71.38 ± 6.14 µM/g in *S. hystrix* [32], 24.81 ± 2.07 µg/mL in *U. pinnatifida* [23] and 4.82 ± 0.11 µM/g in *P. australis* [46], highlighting species-dependent variations in antioxidant potency. *Saccharina wightii* exhibited strong DPPH radical scavenging activity but relatively low FRAP values, reflecting the different mechanisms measured by these assays. DPPH primarily evaluates the ability of compounds to donate hydrogen atoms to neutralize free radicals, whereas FRAP measures the electron-donating capacity to reduce ferric ions. The structural features of *S. wightii* fucoidan such as accessible sulfate groups and hydrogen-donating moieties favor radical scavenging (DPPH) but may not efficiently participate in electron transfer reactions required for FRAP. This demonstrates that antioxidant activity of fucoidans is assay-dependent and influenced by specific structural characteristics rather than total sulfation alone.

Fucoidan extracted from *S. wightii* exhibited strong α-amylase inhibitory activity (IC₅₀ = 0.042 ± 0.02 mg/mL) as compared to the standard drug acarbose (IC₅₀ = 0.108 ± 0.017 mg/mL) as well as *U. pinnatifida* (IC₅₀ = 0.190 ± 0.005 mg/mL) [24] and *A. nodosum* (IC₅₀ = 0.12–4.64 mg/mL) [44]. Comparable inhibitory potential has been reported in studies in *T. ornata* (IC₅₀ = 33.6 ± 0.7 µg/mL) [21] and *S. polycystum* (IC₅₀ = 2.30 ± 0.05 mg/mL) [39]. The pronounced enzyme inhibition observed in *S. wightii* may be attributed to its high sulfate content and molecular configuration, which enhance binding affinity toward the enzyme’s active site.

Similarly, α-D-glucosidase inhibition was most potent in *S. cinereum* (IC₅₀ = 0.22 ± 0.013 mg/mL) comparing of acarbose (IC₅₀ = 1 ± 0.3 mg/mL) [45]. Comparable activities have been documented in *E. maxima* (IC₅₀ = 0.27–0.31 mg/mL) [46], *S. wightii* (IC₅₀ = 132 ± 0.02 µg/mL) [26], *S. polycystum* (IC₅₀ = 3.71 ± 0.04 mg/mL) [39] and *Saccharina japonica* (IC₅₀ = 153.27 ± 22.89 µg/mL) [47]. The present findings also align closely with *U. pinnatifida* (IC₅₀ = 0.137 ± 0.012 mg/mL) [43], suggesting an inhibitory mechanism among brown algal fucoidans. Fucoidan from *S. wightii* selectively inhibited α-amylase, likely due to accessible sulfate groups and flexible backbone structure, whereas *S. cinereum* fucoidan preferentially inhibited α-glucosidase, reflecting a more compact structure and distinct sulfate positioning. These findings highlight that enzyme-specific inhibition is governed by species-dependent fucoidan structural features. Overall, the potent α-amylase and α-glucosidase inhibition in *S. wightii* and *S. cinereum* highlights their strong antidiabetic potential through carbohydrate-hydrolyzing enzyme suppression.

The present study demonstrates the potent anticancer activity of fucoidan derived from *Padina boergesenii* and *Turbinaria conoides*. When compared with the standard anticancer drug doxorubicin (10 µg/mL), which reduced MCF-7 cell viability to 7–9% after 48 h, *P. boergesenii* fucoidan demonstrated comparable cytotoxic potential, while other extracts showed moderate inhibition. *Padina boergesenii* exhibited the highest cytotoxicity despite moderate sulfation, suggesting that cytotoxic activity is influenced by multiple structural features beyond total sulfate content. Factors such as molecular weight, monosaccharide composition, fucose content, branching, and specific sulfate positioning can enhance interactions with cancer cell membranes or intracellular targets, leading to stronger bioactivity. Therefore, moderate overall sulfation does not preclude potent cytotoxic effects if other structural attributes favor biological activity. This aligns with previous reports indicating that structural nuances, not only sulfate density, determine fucoidan’s anticancer potential. Notably, many studies have reported comparable cytotoxic effects. A study by [47] found that 48 h treatment with crude *Undaria pinnatifida* fucoidan (1 mg/mL) left only 2.6%viable MCF-7 cells. Fucoidan from *S. polycystum* inhibited MCF-7 breast cancer cell proliferation (IC₅₀ ≈ 50 ± 0.08 µg/mL) [48]. The cytotoxic effect of fucoidan from *P.boergesenii* on human cervical carcinoma cells demonstrated a significant percentage of inhibition 54% at 40 μg/mL for [28]. Fucoidan-Based Gold Nanoparticles derived from *T. decurrens* and *S. cinereum* exhibited significant activity against HepG2 cells, with IC50 values of 449.5 µg mL − 1 and 337.6 µg mL − 1, respectively [49].

The efficacy of fucoidan is largely governed by its structural characteristics, particularly sulfate content, molecular weight and fucose-to-sulfate ratio which modulate its cellular uptake and interaction with cancer cell receptors [50]. Studies have shown that fucoidan induces both extrinsic and intrinsic apoptotic pathways through activation of caspases-8 9, 3, cytochrome c release and downregulation of anti-apoptotic [51]. In particular, fucoidan has been shown to induce reactive oxygen species (ROS)-mediated apoptosis in human bladder cancer cells by inhibiting telomerase activity and suppressing the PI3K/Akt signaling cascade, leading to programmed cell death [52].

Overall, our data demonstrate that fucoidan’s cytotoxicity toward MCF-7 cells is both species-specific and statistically significant, with extracts from *P. boergesenii* and *T. conoides* showing the most promising activity, approaching the effect of standard doxorubicin. This enhanced activity may arise from apoptotic and oxidative mechanisms associated with higher sulfate substitution. Such ROS-dependent cytotoxicity emphasizes the dual role of fucoidan as both a redox regulator and a signal modulator in cancer suppression.

In addition to the *in vitro* findings, our molecular docking analysis further supports these observations by demonstrating favorable binding affinities of fucoidan toward key apoptosis- and survival-related targets. The predicted interactions with proteins involved in the PI3K/Akt pathway and caspase activation provide a mechanistic rationale for the experimentally observed cytotoxic effects. However, while docking offers valuable insights into potential ligand-protein interactions, these computational predictions require further validation through molecular dynamics simulations, target-specific enzymatic assays, and gene/protein expression studies.

Therefore, future work should focus on integrating advanced *in silico* approaches, including molecular dynamics and binding free energy calculations, with *in vitro* and *in vivo* validation to comprehensively elucidate the structure–activity relationship of fucoidan. Such studies will strengthen the mechanistic understanding of its anticancer potential and support its development as a multi-targeted natural therapeutic agent.

### PCA analysis

**Fig 8:**
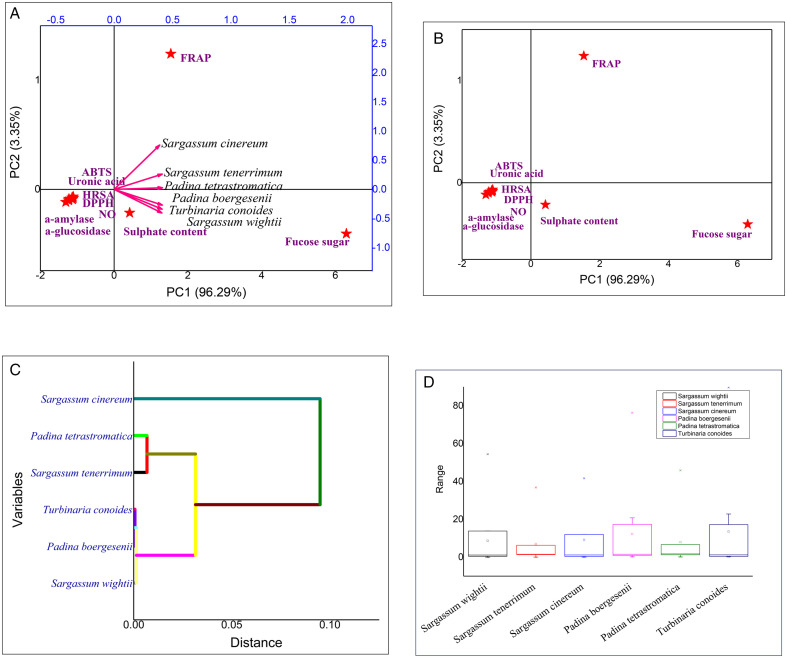
(A) PCA Biplot analysis representing the variation in different bioactivities in fucoidan. **(B)** PCA Score plot analysis representing the variation in different bioactivities in fucoidan. **(C)** Dendrogram showing Agglomerative Hierarchical Clustering. **(D)** Boxplot Analysis.

Principal Component Analysis (PCA) is an advanced multivariate statistical reduction technique that transforms complex datasets into a more interpretable form by reducing multiple variables into a few principal components (PCs) that explain the maximum variance within the data. In this study, PCA and biplot analyses were performed to understand the interrelationship between the antioxidant and enzymatic parameters of six brown seaweed species (**Fig 8A**). The biplot revealed that FRAP activity, fucose, and sulfate content were positively correlated with all *Sargassum* species, whereas the remaining parameters showed negative associations. Among the species, *Padina boergesenii* exhibited the highest fucose content, *Turbinaria conoides* had the maximum sulfate content, and *Sargassum cinereum* showed the strongest FRAP activity. In contrast, ABTS activity displayed the least variance across species.

The score plot (**Fig 8B**) further demonstrated that FRAP and fucose contributed most significantly to data variance, while other variables were moderately correlated. Notably, DPPH IC₅₀ values exhibited a negative correlation with both FRAP activity and fucose content, suggesting that higher antioxidant potential corresponds to lower IC₅₀ values. Similarly, α-amylase inhibitory activity showed a positive correlation with FRAP and fucose, indicating a possible linkage between antioxidant and enzyme inhibitory mechanisms. The clustering of FRAP, fucose, and sulfate along the same principal component suggests that sulfate-rich fucoidan fractions may directly contribute to enhanced antioxidant performance, as the sulfation degree is known to increase electron-donating and radical-scavenging capacity in brown algal polysaccharides. Principal component analysis (PCA) was conducted using bioactivity data exclusively, encompassing the measured biological activity parameters of the fucoidan samples. Due to the functional relationships among these bioactivity variables, they exhibited strong inter-correlations across samples. As a result, the first principal component (PC1) captured the majority of the variance, explaining 96.29% of the total variance. Such a high contribution of PC1 is common in PCA models with a limited set of highly correlated functional variables, where a single dominant factor largely governs the overall biological response. The remaining variance was distributed among the subsequent components (PC2 and higher), which contributed only marginally to the differentiation of samples. It should be noted that this high dominance of PC1 limits the discriminatory power of the PCA. Incorporating structural variables (e.g., molecular weight, branching patterns) in future analyses would likely produce a more informative and discriminating model.

Agglomerative Hierarchical Clustering (AHC) was employed to classify the seaweed species based on their overall biochemical similarity (**Fig 8C**). The dendrogram revealed close clustering among *T. conoides, P. boergesenii,* and *S. wightii,* all of which were characterized by relatively high sulfate and fucose levels, correlating with elevated FRAP and α-amylase inhibitory activities. This grouping highlights a shared biochemical profile driven by sulfate-rich fucoidan composition, which is strongly linked to antioxidant potency. In contrast, S. cinereum appeared as a distinct cluster, reflecting its higher FRAP activity but differing sulfation profile, suggesting that its antioxidant capacity may derive from non-sulfated or polyphenolic components. Meanwhile, *P. tetrastromatica and S. tenerrimum* formed a secondary cluster with lower sulfate and fucose contents, aligning with their comparatively moderate antioxidant responses.

Additionally, the box-and-whisker plot (**Fig 8D**) provided a visual summary of data distribution and variability. The highest degree of variability was observed in *T. conoides*, followed by *P. boergesenii, S. wightii, and S. cinereum, while S. tenerrimum* and *P. tetrastromatica* exhibited the least data dispersion, indicating relatively uniform biochemical profiles among their replicates. Overall, the PCA and AHC results together demonstrate that the sulfate and fucose composition of fucoidans serves as a biochemical determinant influencing both antioxidant and enzyme inhibitory activities across brown seaweed species, confirming the close biochemical–functional correlation among *Sargassum* and related genera.

## Conclusion

This study highlights the structural diversity and potent bioactivity of fucoidan extracted from various brown seaweeds along the Indian coast. Characterization through FT-IR and ^1^H NMR spectroscopy confirmed the presence of functional groups associated with bioactive fucoidan, including sulfate esters and monosaccharides such as fucose and galactose. The composition particularly fucose and sulfate content varied significantly across species, influencing their antioxidant, antidiabetic, and anticancer properties.

Among the studied species, *Sargassum wightii*, *S. cinereum*, and *Padina boergesenii* demonstrated superior biological activities. Fucoidan from *S. wightii* exhibited strong α-amylase and ABTS radical scavenging activity, while *S. cinereum* showed notable DPPH, FRAP, and α-D-glucosidase inhibitory activities. These activities are likely attributed to the favorable sulfate-to-fucose ratios and molecular configurations of the polysaccharides. Additionally, the low sugar and uronic acid content in some species further supports the potential of fucoidan as a functional food component with low glycemic impact.

The findings confirm the therapeutic potential of brown seaweed-derived fucoidan, supporting its application in nutraceuticals, functional foods, and pharmaceuticals. Further research on structure-function relationships and seasonal or geographical variability will strengthen its commercial viability and facilitate targeted bioactive formulation.

## Supporting information

S1 Fig Graphical abstract PlosOne. (DOC)

S1 File Values. (XLSX)

## Abbreviations

DPPH: 2,2-diphenyl-1-picrylhydrazyl
HRSA: Hydroxyl Radical Scavenging Activity
ABTS: 2,2’-azino-bis 3-ethylbenzothiazoline-6-sulfonic acid
NO: Nitric Oxide
FRAP: Ferric Reducing Antioxidant Power Assay

## References

[1] Gupta S, Abu-Ghannam N. Bioactive potential and possible health effects of edible brown seaweeds. Trends in Food Science & Technology. 2011;22(6):315–26. doi: 10.1016/j.tifs.2011.03.011

[2] Choudhary B, Chauhan O, Mishra A. Edible seaweeds: a potential novel source of bioactive metabolites and nutraceuticals with human health benefits. Frontiers in Marine Science. 2021;8:740054.

[3] Wang Y, Xing M, Cao Q, Ji A, Liang H, Song S. Biological Activities of Fucoidan and the Factors Mediating Its Therapeutic Effects: A Review of Recent Studies. Mar Drugs. 2019;17(3):183. doi: 10.3390/md17030183 30897733 PMC6471298

[4] Mensah EO, Kanwugu ON, Panda PK, Adadi P. Marine fucoidans: Structural, extraction, biological activities and their applications in the food industry. Food Hydrocolloids. 2023;142:108784. doi: 10.1016/j.foodhyd.2023.108784

[5] Luthuli S, Wu S, Cheng Y, Zheng X, Wu M, Tong H. Therapeutic Effects of Fucoidan: A Review on Recent Studies. Mar Drugs. 2019;17(9):487. doi: 10.3390/md17090487 31438588 PMC6780838

[6] Fitton HJ, Stringer DS, Park AY, Karpiniec SN. Therapies from Fucoidan: New Developments. Mar Drugs. 2019;17(10):571. doi: 10.3390/md17100571 31601041 PMC6836154

[7] Apostolova E, Lukova P, Baldzhieva A, Katsarov P, Nikolova M, Iliev I, et al. Immunomodulatory and Anti-Inflammatory Effects of Fucoidan: A Review. Polymers (Basel). 2020;12(10):2338. doi: 10.3390/polym12102338 33066186 PMC7602053

[8] Fadel A, Ibrahim HAH, Al-Sodany YM, Bessat M, Abdelsalam M, Amer MS. Prevalence and molecular characterization of Acute hepatopancreatic necrosis disease (AHPND) in cultured white-leg shrimp Litopenaeus vannamei with the fungal bioactive control strategy. Microb Pathog. 2025;203:107450. doi: 10.1016/j.micpath.2025.107450 40054677

[9] Black HS. Oxidative Stress and ROS Link Diabetes and Cancer. JMP. 2024;5(1):96–119. doi: 10.3390/jmp5010007

[10] Agrawal S, Samanta S, Deshmukh SK. The antidiabetic potential of endophytic fungi: Future prospects as therapeutic agents. Biotechnol Appl Biochem. 2022;69(3):1159–65. doi: 10.1002/bab.2192 33998044

[11] Deshmukh SK, Gupta MK, Agrawal S. Antidiabetic Agents from Fungi with Special Reference to α-Glucosidase Inhibitors. Recent Pat Biotechnol. 2023;17(1):24–61. doi: 10.2174/1872208316666220512122439 35549858

[12] Deshmukh SK, Agrawal S, Gupta MK. Fungal Metabolites: A Potential Source of Antidiabetic Agents with Particular Reference to PTP1B Inhibitors. Curr Pharm Biotechnol. 2023;24(8):927–45. doi: 10.2174/1389201023666220506104219 35524660

[13] Zhao Y, Zheng Y, Wang J, Ma S, Yu Y, White WL, et al. Fucoidan Extracted from Undaria pinnatifida: Source for Nutraceuticals/Functional Foods. Mar Drugs. 2018;16(9):321. doi: 10.3390/md16090321 30205616 PMC6164441

[14] DuBois M, Gilles KA, Hamilton JK, Rebers PA, Smith Fred. Colorimetric Method for Determination of Sugars and Related Substances. Anal Chem. 1956;28(3):350–6. doi: 10.1021/ac60111a017

[15] Torres PB, Nagai A, Jara CEP, Santos JP, Chow F, Sdos Santos DYAC. Determination of sulfate in algal polysaccharide samples: a step-by-step protocol using microplate reader. Ocean Coast Res. 2021;69. doi: 10.1590/2675-2824069.21-010pbt

[16] Koh HSA, Lu J, Zhou W. Structure characterization and antioxidant activity of fucoidan isolated from Undaria pinnatifida grown in New Zealand. Carbohydr Polym. 2019;212:178–85. doi: 10.1016/j.carbpol.2019.02.040 30832845

[17] Agrawal S, Barrow CJ, Adholeya A, Deshmukh SK. Unveiling the dermatological potential of marine fungal species components: Antioxidant and inhibitory capacities over tyrosinase. Biotechnol Appl Biochem. 2022;69(3):1252–66. doi: 10.1002/bab.2201 34028084

[18] Prabhakar P, Mukherjee S, Kumar A, Kumar S, Verma DK, Dhara S, et al. Optimization of microwave-assisted extraction (MAE) of key phenolic compounds from pigeon pea (Cajanus cajan L.), their characterization, and measurement of their anti-diabetic and cytotoxic potential. Food Measure. 2023;17(6):5697–720. doi: 10.1007/s11694-023-02082-5

[19] Agrawal S, Nandeibam J, Sarangthem I. Ultrastructural changes in methicillin-resistant Staphylococcus aureus (MRSA) induced by metabolites of thermophilous fungi Acrophialophora levis. PLoS One. 2021;16(10):e0258607. doi: 10.1371/journal.pone.0258607 34648570 PMC8516270

[20] Hifney AF. Industrial optimization of fucoidan extraction from Sargassum sp. and its potential antioxidant and emulsifying activities. Food Hydrocolloids. 2016;54:77–88.

[21] Wang Z, Hsu C, Yin M. Antioxidative characteristics of aqueous and ethanol extracts of glossy privet fruit. Food Chemistry. 2009;112(4):914–8. doi: 10.1016/j.foodchem.2008.06.078

[22] Ikram EHK, Eng KH, Jalil AMM, Ismail A, Idris S, Azlan A, et al. Antioxidant capacity and total phenolic content of Malaysian underutilized fruits. Journal of Food Composition and Analysis. 2009;22(5):388–93. doi: 10.1016/j.jfca.2009.04.001

[23] Prabhakar P, Mukherjee S, Kumar A, Kumar Rout R, Kumar S, Kumar Verma D, et al. In Silico, In Vitro and Ex Vivo Evaluation of the Antihyperglycaemic, Antioxidant and Cytotoxic Properties of Coccinia grandis L. Leaf Extract. Food Technol Biotechnol (Online). 2024;62(2):188–204. doi: 10.17113/ftb.62.02.24.8162 PMC11261651 39045303

[24] Koh HSA, Lu J, Zhou W. Structural Dependence of Sulfated Polysaccharide for Diabetes Management: Fucoidan From Undaria pinnatifida Inhibiting α-Glucosidase More Strongly Than α-Amylase and Amyloglucosidase. Front Pharmacol. 2020;11:831. doi: 10.3389/fphar.2020.00831 32581797 PMC7289976

[25] Kamiloglu S, Sari G, Ozdal T, Capanoglu E. Guidelines for cell viability assays. Food Frontiers. 2020;1(3):332–49. doi: 10.1002/fft2.44

[26] Rupérez P, Ahrazem O, Leal JA. Potential antioxidant capacity of sulfated polysaccharides from the edible marine brown seaweed Fucus vesiculosus. J Agric Food Chem. 2002;50(4):840–5. doi: 10.1021/jf010908o 11829654

[27] Shanthi N, Arumugam P, Murugan M, Sudhakar MP, Arunkumar K. Extraction of Fucoidan from Turbinaria decurrens and the Synthesis of Fucoidan-Coated AgNPs for Anticoagulant Application. ACS Omega. 2021;6(46):30998–1008. doi: 10.1021/acsomega.1c03776 34841142 PMC8613821

[28] Li B, Lu F, Wei X, Zhao R. Fucoidan: structure and bioactivity. Molecules. 2008;13(8):1671–95. doi: 10.3390/molecules13081671 18794778 PMC6245444

[29] Din NAS, Aiemcharoen P, Sermwittayawong D, Lim SJ, Ishak AA, Sofian-Seng N-S, et al. Bioactive properties of fucoidan from Malaysian brown seaweed (Sargassum binderi) with an assessment of its anti-diabetic potential in 3T3-L1 adipocytes. J Food Sci Technol. 2026;63(1):156–67. doi: 10.1007/s13197-025-06410-z 41684468 PMC12891271

[30] Espinosa-Velázquez G, Ramos-de-la-Peña AM, Montanez J, Contreras-Esquivel JC. Rapid physicochemical characterization of innovative fucoidan/fructan powders by ATR-FTIR. Food Sci Biotechnol. 2017;27(2):411–5. doi: 10.1007/s10068-017-0265-1 30263764 PMC6049647

[31] Rajauria G, Ravindran R, Garcia-Vaquero M, Rai DK, Sweeney T, O’Doherty J. Purification and Molecular Characterization of Fucoidan Isolated from Ascophyllum nodosum Brown Seaweed Grown in Ireland. Mar Drugs. 2023;21(5):315. doi: 10.3390/md21050315 37233509 PMC10223938

[32] Webber JL, Benbow NL, Krasowska M, Beattie DA. Formation and enzymatic degradation of poly-l-arginine/fucoidan multilayer films. Colloids Surf B Biointerfaces. 2017;159:468–76. doi: 10.1016/j.colsurfb.2017.08.005 28837896

[33] Antonisamy AJ, Rajendran K. Comparative study on the extraction methods, characterization, and bioactivity of crude fucoidan, a polysaccharide derived from Sargassum ilicifolium. Biochemical Engineering Journal. 2024;209:109398. doi: 10.1016/j.bej.2024.109398

[34] Lakshmanasenthil S, Vinothkumar T, Geetharamani D, Marudhupandi T, Suja G, Sindhu NS. Fucoidan—a novel α-amylase inhibitor from Turbinaria ornata with relevance to NIDDM therapy. Biocatalysis and Agricultural Biotechnology. 2014;3(3):66–70. doi: 10.1016/j.bcab.2014.02.003

[35] Chandía NP, Matsuhiro B. Characterization of a fucoidan from Lessonia vadosa (Phaeophyta) and its anticoagulant and elicitor properties. Int J Biol Macromol. 2008;42(3):235–40. doi: 10.1016/j.ijbiomac.2007.10.023 18054382

[36] Marudhupandi T, Kumar TTA, Senthil SL, Devi KN. In vitro antioxidant properties of fucoidan fractions from Sargassum tenerrimum. Pak J Biol Sci. 2014;17(3):402–7. doi: 10.3923/pjbs.2014.402.407 24897795

[37] Lakshmanan A, Balasubramanian B, Maluventhen V, Malaisamy A, Baskaran R, Liu W-C, et al. Extraction and Characterization of Fucoidan Derived from Sargassum ilicifolium and Its Biomedical Potential with In Silico Molecular Docking. Applied Sciences. 2022;12(24):13010. doi: 10.3390/app122413010

[38] Vinoth Kumar T, Lakshmanasenthil S, Geetharamani D, Marudhupandi T, Suja G, Suganya P. Fucoidan--a α-D-glucosidase inhibitor from Sargassum wightii with relevance to type 2 diabetes mellitus therapy. Int J Biol Macromol. 2015;72:1044–7. doi: 10.1016/j.ijbiomac.2014.10.013 25453283

[39] Obluchinskaya ED, Pozharitskaya ON, Zakharov DV, Flisyuk EV, Terninko II, Generalova YE, et al. The Biochemical Composition and Antioxidant Properties of Fucus vesiculosus from the Arctic Region. Mar Drugs. 2022;20(3):193. doi: 10.3390/md20030193 35323492 PMC8954510

[40] Lim SJ, Wan Aida WM, Maskat MY, Mamot S, Ropien J, Mazita Mohd D. Isolation and antioxidant capacity of fucoidan from selected Malaysian seaweeds. Food Hydrocolloids. 2014;42:280–8. doi: 10.1016/j.foodhyd.2014.03.007

[41] Husni A, Izmi N, Ayunani FZ, Kartini A, Husnayain N, Isnansetyo A. Characteristics and Antioxidant Activity of Fucoidan from Sargassum hystrix: Effect of Extraction Method. Int J Food Sci. 2022;2022:3689724. doi: 10.1155/2022/3689724 35465218 PMC9020993

[42] Kang M-C, Lee H, Choi H-D, Jeon Y-J. Antioxidant properties of a sulfated polysaccharide isolated from an enzymatic digest of Sargassum thunbergii. Int J Biol Macromol. 2019;132:142–9. doi: 10.1016/j.ijbiomac.2019.03.178 30926508

[43] Puhari SSM, Yuvaraj S, Vasudevan V, Ramprasath T, Rajkumar P, Arunkumar K, et al. Isolation and characterization of fucoidan from four brown algae and study of the cardioprotective effect of fucoidan from Sargassum wightii against high glucose-induced oxidative stress in H9c2 cardiomyoblast cells. J Food Biochem. 2022;46(12):e14412. doi: 10.1111/jfbc.14412 36121745

[44] Cholaraj R, Venkatachalam R. Investigation of antioxidant and anticancer potential of fucoidan (in-vitro & in-silico) from brown seaweed Padina boergesenii. Algal Research. 2024;79:103442. doi: 10.1016/j.algal.2024.103442

[45] Costa LS, Fidelis GP, Cordeiro SL, Oliveira RM, Sabry DA, Câmara RBG, et al. Biological activities of sulfated polysaccharides from tropical seaweeds. Biomed Pharmacother. 2010;64(1):21–8. doi: 10.1016/j.biopha.2009.03.005 19766438

[46] Fukuchi A. Evaluation of cytotoxic and antioxidant activity of fucose-containing sulfated polysaccharide from hawaiian marine algae. Interprofessional Journal of Health Sciences. 2017;15(2):15–31.

[47] Mak W. Anti-proliferation potential and content of fucoidan extracted from sporophyll of New Zealand Undaria pinnatifida. Frontiers in Nutrition. 2014;1:9. 25988112 10.3389/fnut.2014.00009 PMC4428450

[48] Fernando IPS, Sanjeewa KKA, Lee HG, Kim H-S, Vaas APJP, De Silva HIC, et al. Fucoidan Purified from Sargassum polycystum Induces Apoptosis through Mitochondria-Mediated Pathway in HL-60 and MCF-7 Cells. Mar Drugs. 2020;18(4):196. doi: 10.3390/md18040196 32276359 PMC7230577

[49] Newehy ASE, Gheda SF, Ismail MM, Aldisi D, Abulmeaty MMA, Elshobary ME. Fucoidan-Based Gold Nanoparticles: Antioxidant and Anticancer Potential from Turbinaria decurrens and Sargassum cinereum. Pharmaceutics. 2025;17(7):826. doi: 10.3390/pharmaceutics17070826 40733037 PMC12299627

[50] Lin Y, Qi X, Liu H, Xue K, Xu S, Tian Z. The anti-cancer effects of fucoidan: a review of both in vivo and in vitro investigations. Cancer Cell Int. 2020;20:154. doi: 10.1186/s12935-020-01233-8 32410882 PMC7206694

[51] Sajadimajd S, Momtaz S, Haratipour P, El-Senduny FF, Panah AI, Navabi J, et al. Molecular Mechanisms Underlying Cancer Preventive and Therapeutic Potential of Algal Polysaccharides. Curr Pharm Des. 2019;25(11):1210–35. doi: 10.2174/1381612825666190425155126 31465281

[52] Han MH, Lee D-S, Jeong J-W, Hong S-H, Choi I-W, Cha H-J, et al. Fucoidan Induces ROS-Dependent Apoptosis in 5637 Human Bladder Cancer Cells by Downregulating Telomerase Activity via Inactivation of the PI3K/Akt Signaling Pathway. Drug Dev Res. 2017;78(1):37–48. doi: 10.1002/ddr.21367 27654302

