# Supplementary figures and images for: Multifaceted bioactivity of brown seaweed-derived fucoidan from the Indian Coastline: A natural health product candidate
Source: PLoS One. 2026 Apr 7;21(4):e0346712. doi: 10.1371/journal.pone.0346712 (PMC13056200; doi:10.1371/journal.pone.0346712)

**
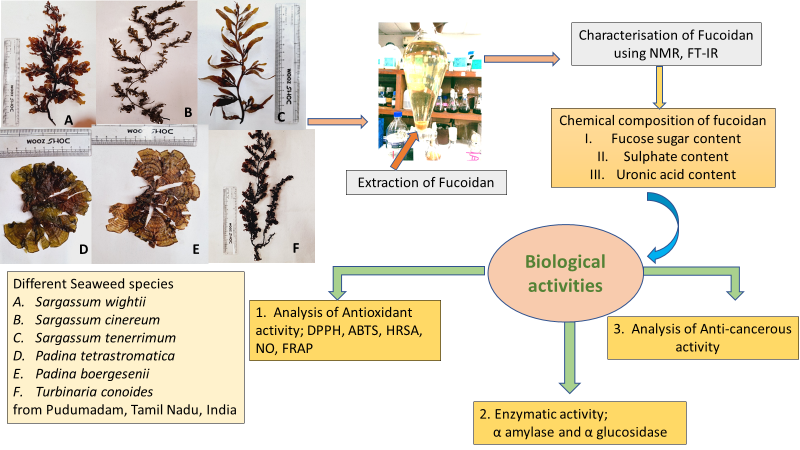
**

Graphical Abstract showing Fucoidan and its various activities

Supplement: S1 Fig — (DOC) [file pone.0346712.s001.doc]
